# A Highly Sensitive Pressure-Sensing Array for Blood Pressure Estimation Assisted by Machine-Learning Techniques

**DOI:** 10.3390/s19040848

**Published:** 2019-02-19

**Authors:** Kuan-Hua Huang, Fu Tan, Tzung-Dau Wang, Yao-Joe Yang

**Affiliations:** 1 Department of Mechanical Engineering, National Taiwan University, Taipei 10617, Taiwan; hank831030@mems.me.ntu.edu.tw (K.-H.H.); tanfu@mems.me.ntu.edu.tw (F.T.); 2 Department of Medicine, National Taiwan University, Taipei 10617, Taiwan; tdwang@ntu.edu.tw; 3 National Taiwan University Hospital, Taipei 10002, Taiwan

**Keywords:** microstructure, polymer sensor, pulse-wave monitoring, machine learning technique, blood-pressure estimation

## Abstract

This work describes the development of a pressure-sensing array for noninvasive continuous blood pulse-wave monitoring. The sensing elements comprise a conductive polymer film and interdigital electrodes patterned on a flexible Parylene C substrate. The polymer film was patterned with microdome structures to enhance the acuteness of pressure sensing. The proposed device uses three pressure-sensing elements in a linear array, which greatly facilitates the blood pulse-wave measurement. The device exhibits high sensitivity (−0.533 kPa^−1^) and a fast dynamic response. Furthermore, various machine-learning algorithms, including random forest regression (RFR), gradient-boosting regression (GBR), and adaptive boosting regression (ABR), were employed for estimating systolic blood pressure (SBP) and diastolic blood pressure (DBP) from the measured pulse-wave signals. Among these algorithms, the RFR-based method gave the best performance, with the coefficients of determination for the reference and estimated blood pressures being R^2^ = 0.871 for SBP and R^2^ = 0.794 for DBP, respectively.

## 1. Introduction

Various recent studies in epidemiology have reported that hypertension is one of the most common causes of cardiovascular disease [1,2], and that blood pressure is a critical physiological parameter in the clinical diagnosis and treatment of hypertension [3]. Also, it has been reported that variability in blood pressure is a prognostic indicator for hypertension [4]. Measuring blood pressure by using cuff sphygmomanometers is the standard practice in clinical diagnosis. This method not only provides fundamental quantitative information on the arterial pressure of patients, but is also useful in the preliminary estimation of cardiovascular risk. However, cuff sphygmomanometers are not suitable for continuous beat-to-beat blood pressure measurements, due to the periodic inflation and deflation of the cuff during operation. 

An invasive approach is capable of accurately and continuously measuring blood pressure [5,6]. The typical invasive method requires an intravascular cannula needle to be inserted into a vein. An electronic transducer is integrated into the needle, and the blood pressure is measured directly by the transducer [7]. Additionally, several novel implantable devices for monitoring blood pressure have been reported [8]. In [9], the development of a wireless, ambulatory, continuous blood-pressure monitoring system using surface acoustic-wave technology was presented. The device includes a pressure sensor embedded with resonators on a crystalline quartz wafer. Theodor et al. proposed an implantable continuous blood-pressure monitoring device fabricated on a flexible substrate with accelerometers and a wireless transmission unit [10]. In [11], a passive wireless sensing device integrated with a microfabricated inductor and a capacitor was presented. In this device, the blood pressure changes the capacitance, and the capacitance change can be detected and transmitted by using the impedance-phase-dip technique. Although the invasive approach is capable of continuously and directly measuring blood pressure, it is still undesirable under many circumstances, due to several risks, such as injury to the vein, bleeding, and patient discomfort.

In recent years, research on noninvasive blood-pressure measurement has drawn wide attention. Various highly sensitive pressure sensors have been reported for continuously measuring blood pulse waves [12,13,14,15]. Sekine et al. proposed a ferroelectric-polymer flexible sensor for monitoring the pulse wave on the skin [16]. The fully printed, wearable sensor possesses the advantages of high sensitivity, rapid response, and good stability. In [17], a pressure sensor fabricated with an ultrathin inorganic piezoelectric material on elastomer substrates is proposed. This device is capable of low hysteresis measurements of pressure on the skin. Blood-flow pressure measurement of near-surface arteries has also been demonstrated. Yang et al. proposed a self-powered bionic membrane sensor for continuous and noninvasive monitoring of arterial pulse waves [18]. The device is lightweight, wearable, and inexpensive. Furthermore, Zang et al. proposed suspended gate organic thin-film transistors for pressure detection [19]. The proposed flexible transistor is of ultra-high sensitivity, and is capable of the real-time detection of wrist pulses. 

The present work proposes a pressure-sensing array for noninvasive continuous blood pulse-wave monitoring. The sensing elements comprise a polydimethylsiloxane (PDMS) conductive polymer film, and three pairs of interdigital electrodes patterned onto a flexible Parylene C substrate. The polymer film has been patterned with microdome structures for enhancing the acuteness of pressure sensing [20]. The proposed device was designed as a linear-array configuration, in which three pressure-sensing elements are employed. This array design may greatly facilitate the measurement of blood pulse waves. Furthermore, various machine-learning algorithms, including random forest regression (RFR), gradient-boosting regression (GBR), and adaptive boosting regression (ABR) were employed for estimating the systolic and diastolic blood pressure from pulse-wave signals. The reminder of the paper is arranged as follows. Section 2 illustrates the device design and the working principle of the pressure-sensing array. Section 3 explains the details of the fabrication process. Section 4 discusses the device’s characterization and the estimated blood-pressure results, while Section 5 concludes this study.

## 2. Device Design and Working Principles

**Figure 1:**
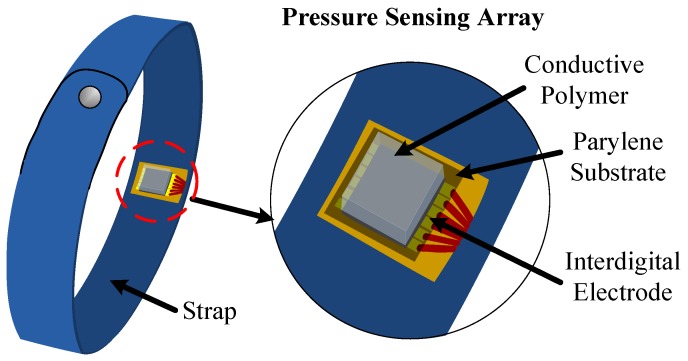
The schematic of the proposed device.

**Figure 2:**
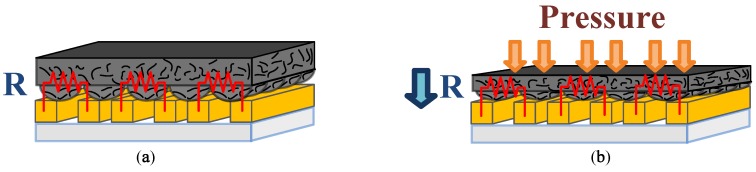
Working principle of the proposed device. (**a**) The initial shape of the conductive polymer. (**b**) The microdome structures deform as external pressure is applied.

A schematic of the proposed blood-pressure monitoring system is shown in Figure 1. The pressure sensing array comprises a conductive polymer thin film and three pairs of interdigital electrodes patterned onto a flexible Parylene C substrate; it is packaged using thin Surlyn films and mounted on a strap. The conductive polymer film is patterned with microdome structures to enhance the sensitivity of the proposed device. Figure 2 illustrates the working principle of the device’s pressure-sensing element. As shown in Figure 2b, the microdome structures on the conductive polymer film deform when external pressure is applied, and, consequently, the contact area between the conductive polymer and the interdigital electrodes rapidly increases, which results in a sharp decrease in the contact resistance. This type of piezoresistive effect exhibits a much higher sensitivity than the typical bulk conductive polymer materials that employ the percolation theory [21]. 

**Figure 3:**
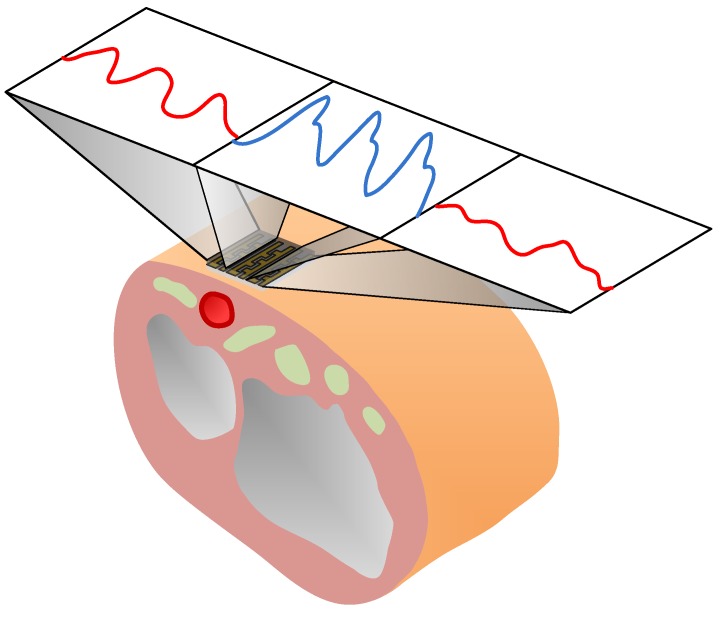
The operational scenario of the proposed device.

Figure 3 shows the advantage of using the array-type configuration for measuring the blood pulse wave, using the tonometry method. In general, it is difficult and troublesome to precisely align the sensor with the radial artery to capture high-quality signals [22]. As a result, in this work, the proposed device uses three pressure-sensing elements in a linear array. During pulse-wave measurements, at least one of the channels, which are positioned directly above the radial artery, gives the best signal quality, provided that the sensing array fully covers the skin above the radial artery. This array configuration may greatly facilitate blood pulse-wave measurements.

## 3. Fabrication

### 3.1. Fabrication of the Conductive Polymer

The proposed conductive polymer was fabricated by dispersing multi-wall carbon nanotubes (MWCNTs) in polydimethylsiloxane (PDMS) prepolymer. The prepolymer was prepared as follows: First, the PDMS prepolymer (Sylgard 184 A, Dow Corning Corp., Midland, MI, USA) was mixed with hexane at a ratio of 4:1. Next, the solution of MWCNTs with diameters of about 20 nm and lengths ranging from 10 to 50 μm was dispersed in the mixture at a concentration of 6% by weight. The prepolymer mixture was thoroughly stirred for 6 hr by a magnetic stirrer. The mixing performance was greatly improved by the hexane, which served as a dispersant. The PDMS-curing agent (Sylgard 184 B, Dow Corning Corp., Midland, MI, USA) was then added to the prepolymer at a ratio of 1:10, and stirred for 20 m. Finally, the prepolymer mixture was degassed in a vacuum chamber for 30 m to evaporate the solvent, readying it for the subsequent lithography process.

**Figure 4:**
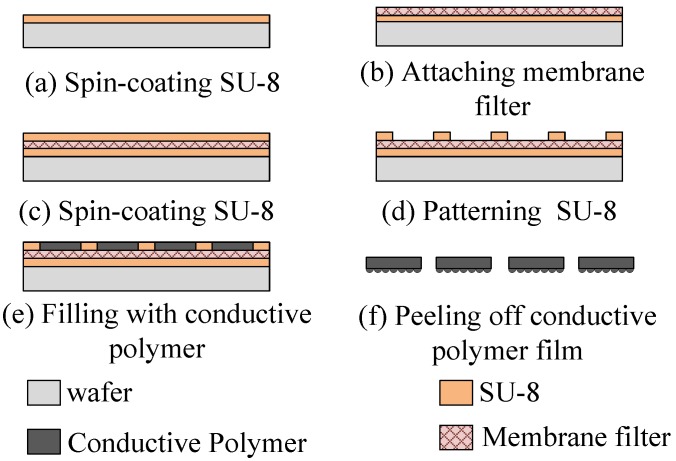
Fabrication process of the conductive polymer.

Figure 4 shows the process of patterning microdome structures on the conductive polymer surface. First, a layer of SU-8 photoresist (SU-8 2050, MicroChem Corp., Westborough, MA, USA) with a thickness of 200 μm was spin-coated onto a glass handling wafer (Figure 4a). A nylon-membrane filter, which served as the mold for the microstructures, was then placed on the photoresist (Figure 4b). Another layer of SU-8 photoresist, with a thickness of 200 μm, was spin-coated onto the membrane filter (Figure 4c), and then the photoresist was patterned to define the dimensions of the conductive polymer after a standard lithography process (Figure 4d). Subsequently, the prepared MWCNT/PDMS composite was filled into the SU-8 trenches (Figure 4e) and cured at 95 °C for 6 h. Finally, after removal of the SU-8 photoresist, the conductive polymer was peeled from the nylon-membrane filter (Figure 4f).

### 3.2. Device Fabrication

**Figure 5:**
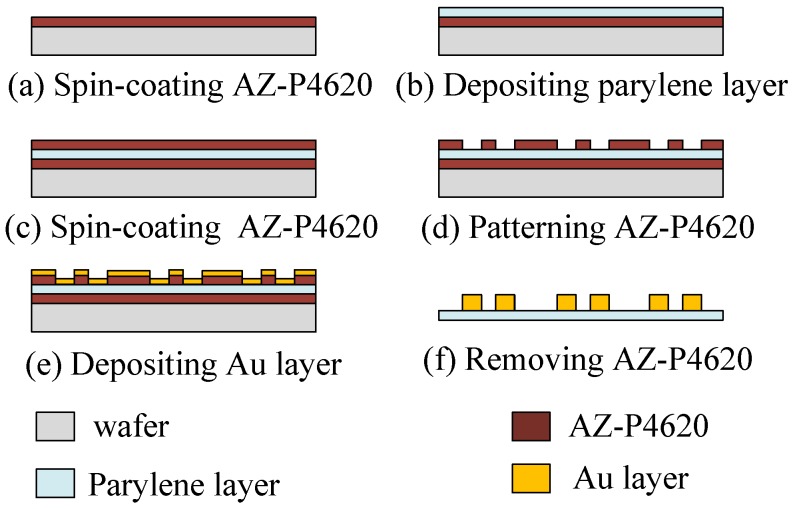
Fabrication process of the interdigital electrode pairs.

The fabrication of the flexible interdigital electrodes is described in Figure 5. First, a sacrificial layer of AZ-P4620 photoresist (AZ-P4620; MicroChem Corp., Westborough, MA, USA) of 3 μm in thickness was spin-coated on a glass wafer (Figure 5a). Next, a 30 μm Parylene C film was deposited onto the sacrificial layer as the flexible substrate for the electrode pairs (Figure 5b). Another layer of 3 μm AZ-P4620 photoresist was then spin-coated onto the layer of Parylene C (Figure 5c), and the interdigital shapes were transferred to the photoresist after a standard lithography process (Figure 5d). Next, a 300 μm Au layer was deposited onto the wafer (Figure 5e). After removal of the AZ-P4620 layer, the flexible electrode pairs were lifted off the handling wafer (Figure 5f).

**Figure 6:**
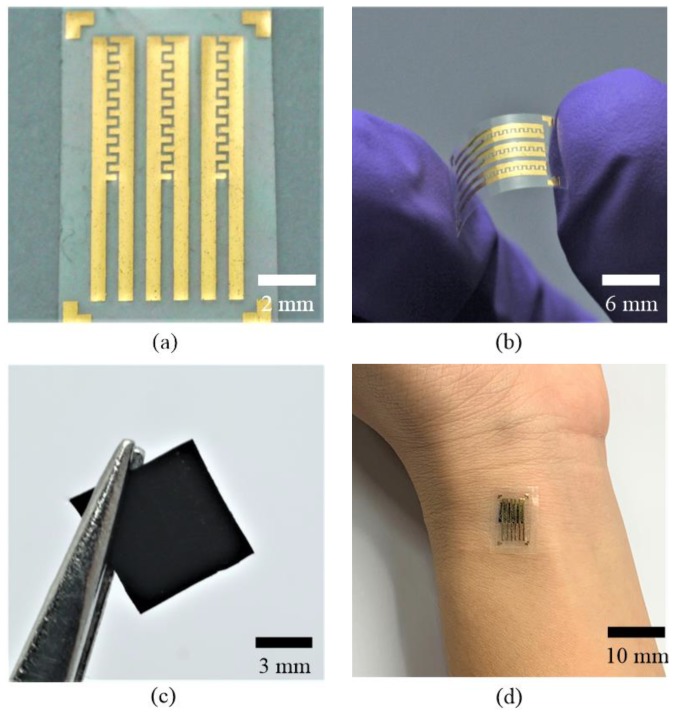
The fabricated results. (**a**) Fabricated electrode pairs. (**b**) The electrode pairs with a flexible substrate. (**c**) Fabricated conductive polymer thin film. (**d**) The assembled device used for blood pulse wave monitoring.

**Figure 7:**
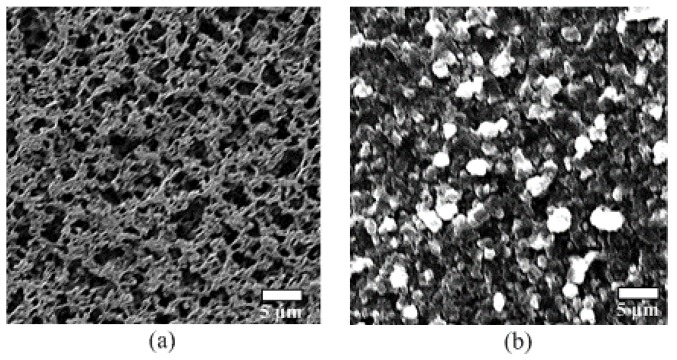
The SEM pictures. (**a**) Nylon membrane filter. (**b**) Conductive polymer.

Figure 6a,b shows the fabricated interdigital electrode pairs on the flexible Parylene C substrate. The finger width of the electrodes is 300 μm, and the gap between the fingers is 250 μm. The planar dimensions of each electrode pair are 1.5 mm × 10 mm, and the thickness of the electrodes is 0.3 μm. Figure 6c shows the fabricated conductive polymer film (6 mm × 6 mm × 200 μm), and Figure 6d pictures the assembled device. Figure 7a shows a scanning electron microscopy (SEM) image of the nylon-membrane filter. As shown in the picture, numerous tiny pores are distributed randomly on the surface of the membrane, which serves as the mold for transferring the microdome structures onto the conductive polymer film. Figure 7b shows an SEM image of the conductive polymer patterned with uniformly distributed microdome structures, with an average diameter of about 3 μm.

## 4. Discussion

### 4.1. Device Characterization

**Figure 8:**
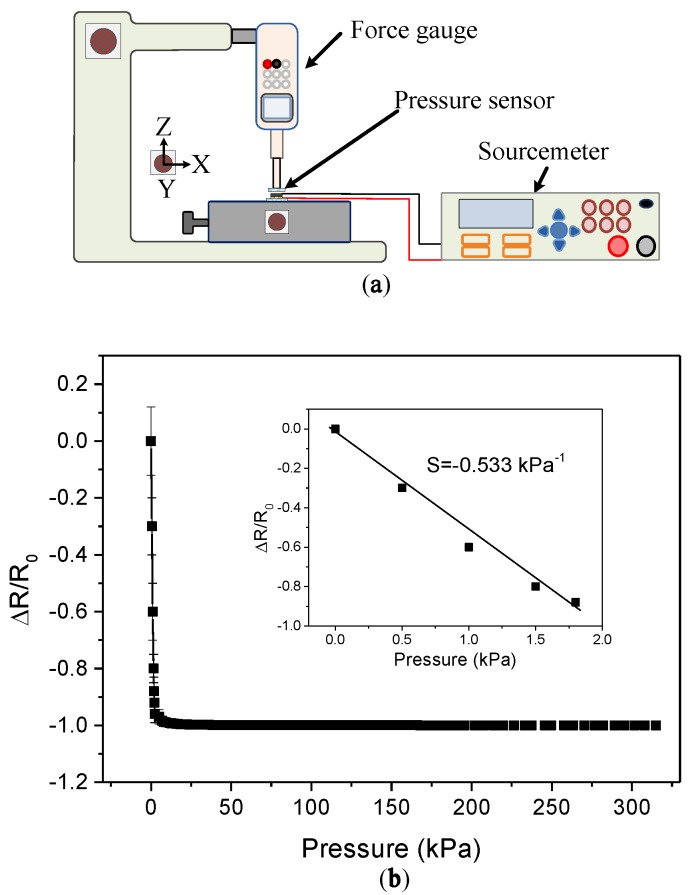
(**a**) Measurement setup for the pressure-sensing array with pressure force. (**b**) The measured relationship between the pressure and the change in resistance.

Figure 8a shows the experimental setup for measuring the piezoresistive characteristics of the sensing element. The force applied to the sensing element was measured by a force gauge (HF-1; ALGOL Engineering Corp., Taoyuan, Taiwan), which was installed onto a vertical translational stage. The maximum resolution of the force gauge was 1 mN. A polymethylmethacrylate (PMMA) rod of 6 mm in diameter was glued to the sensing head of the force gauge. As the stage moved downward, the PMMA rod contacted the sensing element, and the resistance change of the sensing element was measured by a source meter (Model 2400; Keithley Instruments Corp., Solon, OH, USA). Figure 8b shows the results of the pressure-resistance measurements of the proposed device. As light pressure (i.e., below 2 kPa) was applied, the resistance changed significantly because the contact area between the conductive polymer and the electrode pair increased rapidly, due to the deformation of the microdomes, which resulted in a sharp decrease in tunneling resistance. This result indicates that the pressure sensor exhibits a high sensitivity (−0.533 kPa^−1^) at a low pressure range (below 2 kPa). However, when the applied pressure exceeded around 10 kPa, the resistance almost stopped decreasing, which may be due to the fact that the contact area between the microdome structures and the electrode pair reached a steady value.

**Figure 9:**
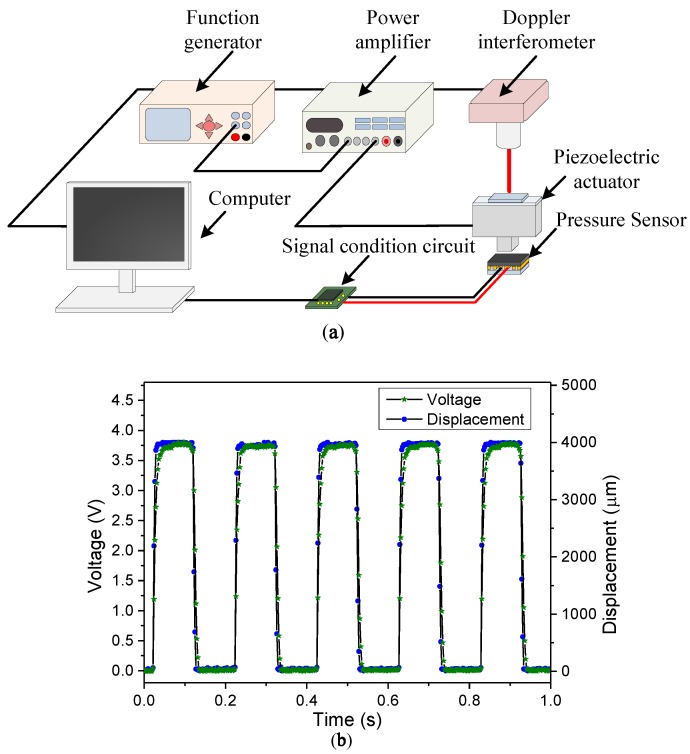
(**a**) Experimental setup for measuring the dynamic response of the sensing element. (**b**) Transient response of the sensing response.

The dynamic response of the proposed sensing element was also measured and discussed. The experimental setup is shown in Figure 9a. A piezoelectric actuator (P-621.20 L, Physik Instruments Corp., Karlsruhe, Germany) was used to generate a displacement input for periodically pressing and releasing the sensing element. The actuator was driven by a power amplifier, which was connected to a function generator supplied with square wave signals. A PMMA block, 3 mm × 3 mm in cross-section, was attached to the head of the piezoelectric actuator. The transient motion of the piezoelectric actuator was measured by using a laser Doppler interferometer. Furthermore, the transient resistance variation of the sensing element was converted into a voltage output by a signal-conditioning circuit, which was connected to a PC equipped with a data-acquisition card. Figure 9b shows the measured transient responses. As shown in the figure, the responses of the sensing element slightly lagged behind the displacement input of the piezoelectric actuator by approximately 12 ms during the pressing–releasing cycles.

**Figure 10:**
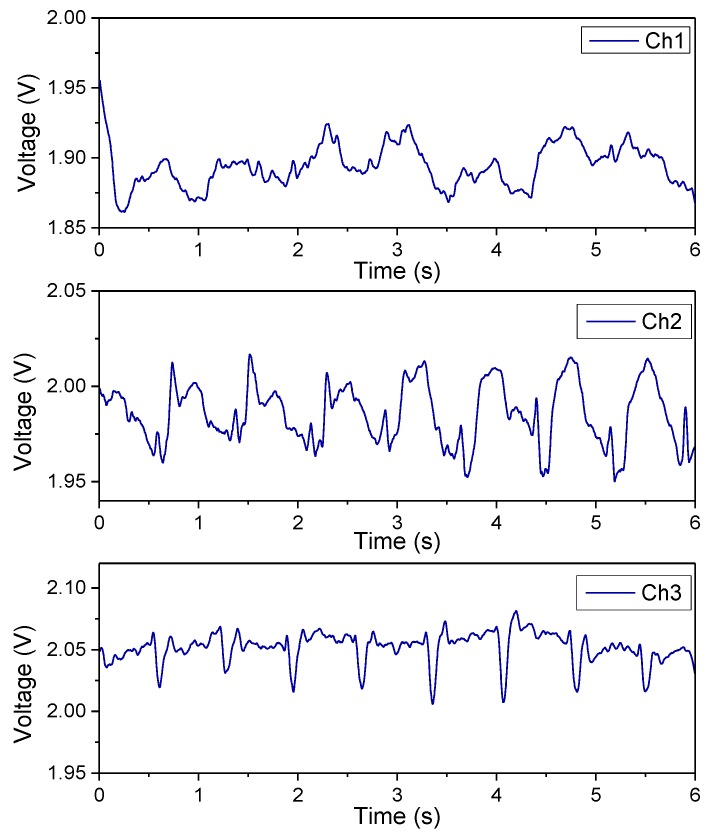
Measured blood pulse wave signals by the 3 × 1 tactile sensing array.

The continuous blood pulse-wave signals measured by the proposed sensing array are presented in Figure 10. The results indicate that Channel 2 gave the highest quality signals, with the most distinguishable peaks and the least noise, because the sensing element of Channel 2 was positioned directly above the radial artery. By contrast, the other channels gave either degraded or unclear blood pulse-waves signals. By using simple signal-processing techniques, the channel that gave the best quality signals could easily be identified.

### 4.2. Blood-Pressure Estimation using Machine Learning Algorithms

**Figure 11:**
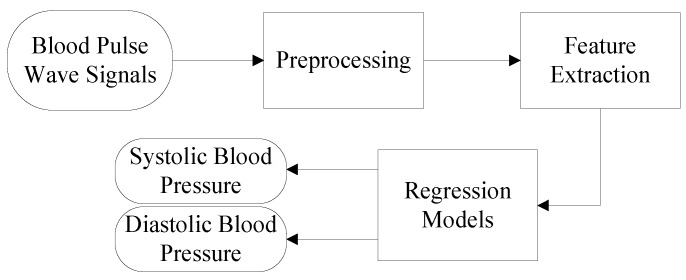
Block diagram of the blood pressure estimation process by machine-learning techniques.

Figure 11 shows the basic block diagram of the blood-pressure estimation process using the proposed device, including five major steps: (1) acquiring blood pulse-wave signals using the proposed pressure sensor, (2) processing the pulse-wave signals by removing baseline wandering, high-frequency noise, and artifacts, (3) extracting the characteristic features from the processed pulse-wave signals, (4) estimating blood pressure by using three regression models, and, finally, (5) analyzing the estimated results statistically. The following paragraphs describe these steps.

**Figure 12:**
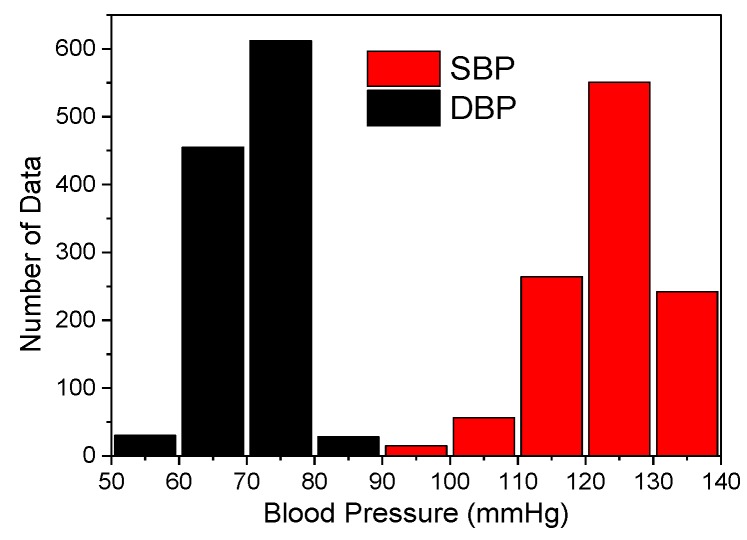
Histogram of the measured signal database.

In this study, the proposed device obtained blood pulse-wave signals from 23 participants. In addition, the reference blood pressures were measured simultaneously using a cuff sphygmomanometer (HEM-7310, OMRON Industrial Automation Japan, Japan). Hence, the measured pulse wave signals corresponded to the simultaneously measured systolic blood pressure (SBP) and diastolic blood pressure (DBP). Figure 12 shows the histogram of the measured database. A total of 1128 datasets (i.e., measured blood pulse wave signals and the reference blood pressures measured by a cuff sphygmomanometer) were obtained. In the datasets, the SBP was in a range of 98 to 138 mmHg, and the DBP was in a range of 58 to 81. 

**Figure 13:**
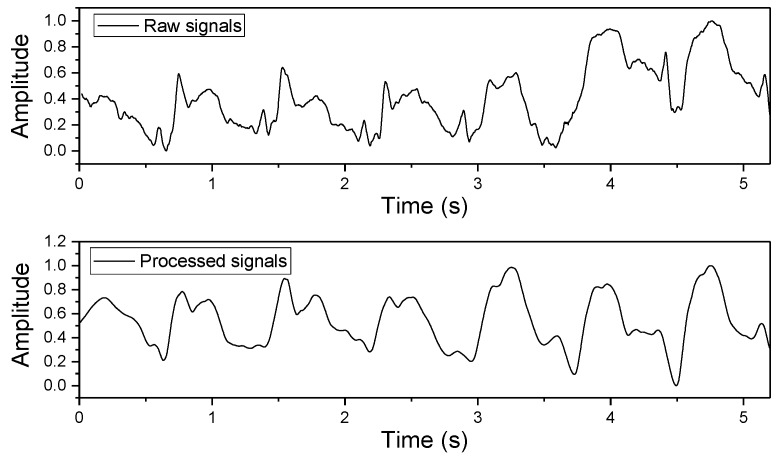
Pulse wave signals before and after Hilbert–Huang transform (HHT) signal preprocessing.

**Figure 14:**
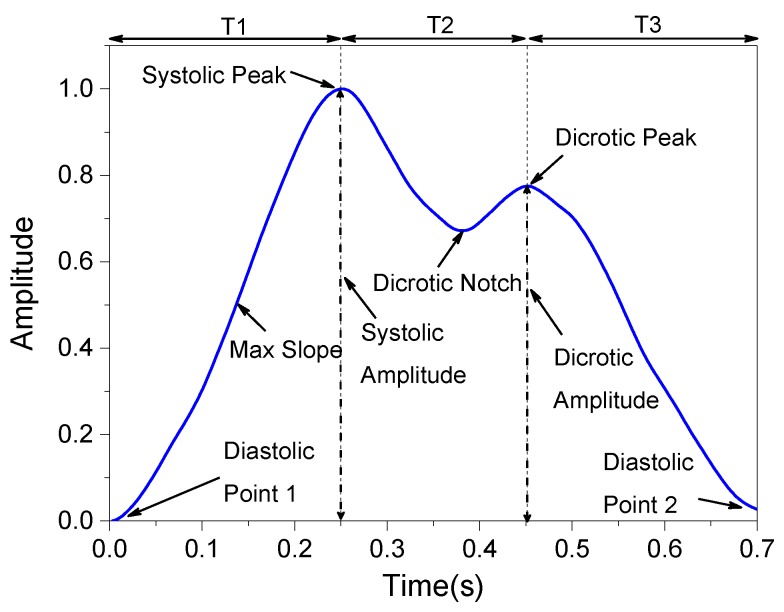
Extraction of blood pulse wave signal features.

In order to remove noise and artifacts from the raw pulse-wave signals, the Hilbert–Huang transform (HHT) method was employed to process the data [23,24]. In general, the HHT includes two steps. In the first, the intrinsic mode functions (IMFs) are generated by using the empirical mode decomposition procedure. In the second, the new wave signals are reconstructed by ignoring the IMFs corresponding to noise parts. The comparison of pulse-wave signals before and after the HHT preprocessing is shown in Figure 13. Next, characteristic features related to blood pressure were are from each pulse wave [25], such as the systolic peak, dicrotic peak, diastolic points, max slope, augmentation index, heart rate, and some important time-interval information. Figure 14 and Table 1 show the definitions of the extracted features used in this work.

The processed pulse-wave signals (data) were split into two sets: 80% of the data were included in the dataset for training the regression models (i.e., the training data), and 20% of the data were included in the dataset for testing the regression models (i.e., the testing data) in estimating the target SBP and DBP values. In this work, three blood-pressure estimation models were constructed, through three distinct regression algorithms, including RFR [26], GBR [27], and ABR [28,29]. These algorithms are briefly introduced as follows: RFR is an ensemble learning method that gives a final prediction by averaging the predictions from many weak learners. In order to achieve a low bias in prediction, each weak learner is trained on a random sample of the training dataset. GBR is a learning procedure that fits new models to give a better estimation of the response variable. This algorithm constructs the new base-learners to be closely correlated with the negative gradient of the loss function, evaluated by using the training dataset. ABR is a method that generates a prediction by combining the outputs of many weak learners into a weighted sum. Although each weak learner is insufficient for accurate prediction in many applications, their weighted sum is comparable to the result of using strong learners. Based on these algorithms, the models for blood pressure prediction in this work were trained using the codes in the Scikit-learn library [30].

**Figure 15:**
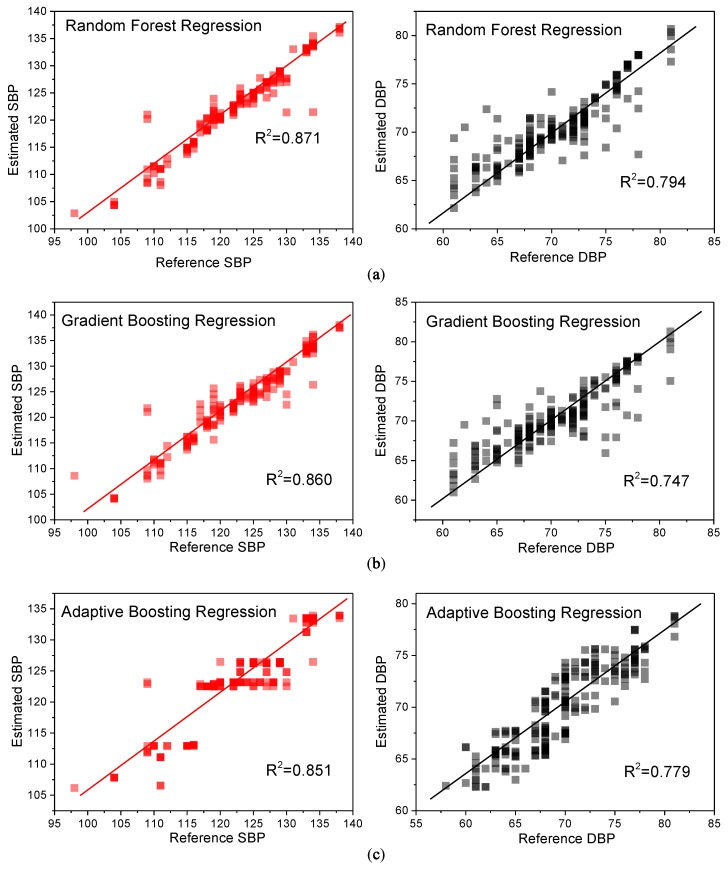
Systolic blood pressure- and diastolic blood pressure-estimated results with three different regression algorithms: (**a**) Random Forest Regression (**b**) Gradient Boosting Regression, and (**c**) Adaptive Boosting Regression.

Figure 15 shows the correlation analysis between the reference blood pressures and the estimated blood pressures. In the figure, the estimated SBP and DBP were calculated from the testing data by using the regression models which were trained by using the training data. Also, the reference blood pressures were measured by using a cuff sphygmomanometer. For the RFR model, the R^2^ between the estimated SBP and the cuff-based SBP was 0.871, while the R^2^ between the estimated DBP and the cuff-based DBP was 0.794. Both R^2^ values (i.e., SBP and DBP) of the RFR model were highest when compared with those of the other two models.

**Figure 16:**
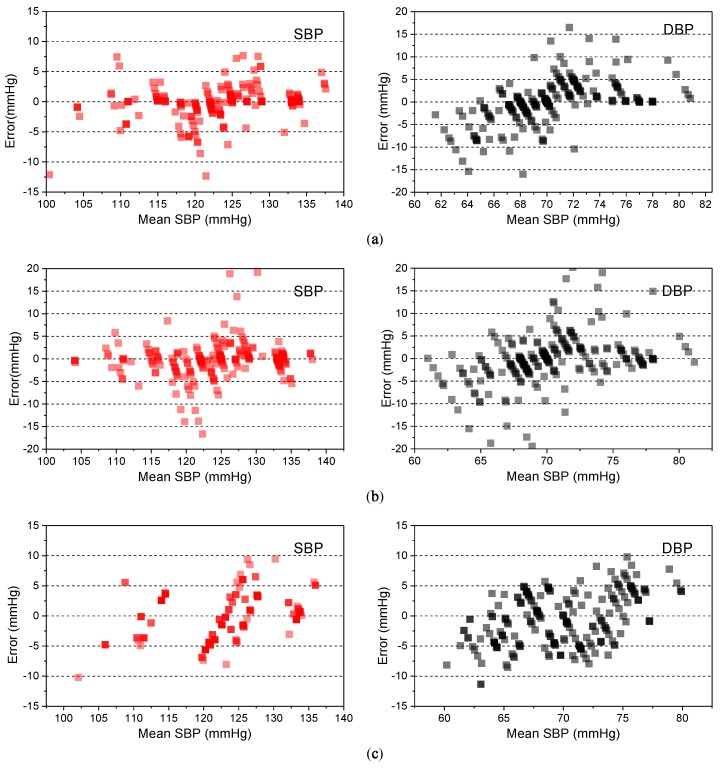
Bland–Altman plots for systolic blood pressure and diastolic blood pressure estimation with three different regression algorithms: (**a**) Random Forest Regression, (**b**) Gradient Boosting Regression, and (**c**) Adaptive Boosting Regression.

Figure 16 shows the Bland–Altman plots for the targets of SBP and DBP, created by using the estimation models generated by the regression algorithms. About 80% of the estimated SBPs and 70% of the estimated DBPs were within the error lines of 5 mmHg. Also, about 3% of the estimated SBPs and 5% of the estimated DBPs were beyond the error lines of 15 mmHg. Table 2 and Table 3 show the comparison between the proposed approaches and other approaches for blood-pressure estimation. As shown in the tables, the RFR-based algorithm gives a better accuracy than the other models, for blood-pressure estimation.

## 5. Conclusions

In this work, a pressure-sensing array for noninvasive continuous blood pulse-wave monitoring is presented. The propose device features a linear-array configuration in which three pressure-sensing elements are utilized. Each element comprises a conductive polymer film and an interdigital electrode pair on a flexible substrate. Microdome structures are patterned on the polymer film for enhancing the pressure-sensing sensitivity. The proposed array design greatly facilitates the measurement of blood pulse waves. The realized device exhibits high sensitivity (−0.533 kPa^−1^) and a fast dynamic response. Furthermore, three distinct machine-learning algorithms were employed to estimate the systolic and diastolic blood pressure from the measured pulse-wave signals. A performance comparison of the algorithms is provided.

## Figures and Tables

**Table 1:** Definitions of the selected features of the measured pulse waves.

Features	Definitions
Heart Rate	The measured peak-to-peak time interval of pulse wave signals.
Systolic Peak	The first peak in the pulse waveform.
Dicrotic Peak	The secondary peak in the pulse waveform.
Diastolic Point 1	The first Diastolic value.
Diastolic Point 2	The second Diastolic value.
Dicrotic Notch	The notch point of the pulse waveform.
Max Slope	The max slope between the Diastolic Point 1 and the Systolic Peak.
Augmentation Index	The amplitude ratio of the Systolic peak and the Dicrotic Peak.
T1	Time interval between the Diastolic Point 1 and the Systolic Peak.
T2	Time interval between the Systolic Peak and the Dicrotic Peak.
T3	Time interval between the Dicrotic Peak and the Diastolic Point 2.

**Table 2:** Comparison of the accuracy of the systolic blood pressure (SBP)-estimated results by three different regression models and other publications.

SBP	<5 mmHg	<10 mmHg	<15 mmHg
Random Forest Regression(RFR)	88.9%	97.4%	98.2%
Gradient Boosting Regression(GBR)	87.7%	95.2%	97.4%
Adaptive Boosting Regression(ABR)	84.1%	93.6%	98.1%
Liu et al. [31]	50.1%	81.2%	94.8%
Mohammad et al. [32]	34.1%	56.5%	72.7%

**Table 3:** Comparison of accuracy of the diastolic blood pressure(DBP)-estimated results by three different regression models and other publications.

DBP	<5 mmHg	<10 mmHg	<15 mmHg
Random Forest Regression(RFR)	75.8%	93.0%	96.9%
Gradient Boosting Regression(GBR)	77.5%	92.5%	96.0%
Adaptive Boosting Regression(ABR)	76.2%	90.9%	93.1%
Liu et al. [31]	64.5%	93.4%	98.8%
Mohammad et al. [32]	62.7%	87.1%	95.7%
